# Psychometric properties and measurement invariance of the 7-item game addiction scale (GAS) among Chinese college students

**DOI:** 10.1186/s12888-020-02830-7

**Published:** 2020-10-02

**Authors:** Yujie Liu, Qian Wang, Min Jou, Baohong Wang, Yang An, Zifan Li

**Affiliations:** 1 School of Public Health, Shanghai Jiao Tong University School of Medicine, No 227 S Chongqing Road, Shanghai, 200025 China; 2 Department of Industrial Education, National Taiwan Normal University, Taipei, Taiwan; 3 Shanghai Jiao Tong University School of Medicine, College of Clinical Medicine, Shanghai, China; 4 School of Social Development and Public Policy, Fudan University, Shanghai, China

**Keywords:** Internet gaming disorder, College students, PSQI, SCL-90-R, Internet addiction, Measurement invariance

## Abstract

**Background:**

The 7-item Gaming Addiction Scale (GAS) has been used as a screening tool for addictive game use worldwide, and this study aimed to examine its psychometric properties and measurement invariance among college students in China.

**Methods:**

Full-time students from multiple colleges in China were recruited. A total of 1040 completed questionnaires were used in the final analysis. Reliability of the GAS was assessed by internal consistency and split-half reliability. Validity of the GAS was assessed by structural validity, convergent validity, discriminant validity, and concurrent validity. A series of Multigroup Confirmatory Factor Analysis (MG-CFA) were conducted to test and establish measurement invariance across gender, class standing, family income and parental educational level.

**Results:**

Exploratory factor analysis revealed a unidimensional structure of the GAS. The GAS exhibited excellent internal consistency (Cronbach’s α = 0.951, theta coefficient = 0.953, omega coefficient = 0.959) and structural validity (χ^2^ /df = 0.877 (*p* < 0.05), CFI = 0.999, TIL = 0.996, RMSEA =0.000). Concurrent validity of the GAS was confirmed by its correlation with problematic internet use, sleep quality, nine dimensions of psychiatric symptoms, and substance use. The GAS also demonstrated measurement invariance across father’s educational level (Δ**χ**2 (df) = 19.128 (12), ΔCFI = − 0.009, ΔRMSEA = 0.010 for weak factorial model; Δ**χ**2 (df) = 50.109 (42), ΔCFI = − 0.010, ΔRMSEA = 0.007 for strict factorial model.) and mother’s educational level (Δ**χ**2 (df) = 6.679 (12), ΔCFI = 0.007, ΔRMSEA = − 0.010 for weak factorial model; Δ**χ**2 (df) =49.131 (42), ΔCFI = − 0.009, ΔRMSEA = − 0.004 for strict factorial model), as well as partial measurement invariance across gender (except for item 2), class standing (except for item 7) and family income (except for item 5).

**Conclusions:**

The Chinese version of the 7-item GAS can be an adequate assessment tool to assess internet gaming disorder among the college student population in China.

## Background

Internet gaming disorder (IGD) has increasingly become an internationally recognized behavioral addiction, constituting a growing concern worldwide including in China. Its inclusion in the fifth edition of the Diagnostic and Statistical Manual of Mental Disorders (DSM-V [1];) has garnered considerable attention from researchers worldwide. The DSM-V clearly defines the diagnostic criteria for IGD, requiring at least 5 out of 9 symptoms (preoccupation, tolerance, escape, withdrawal, persistence, conflict, problems, deception, and displacement) to be present for at least 12 months. Prevalence of IGD varied across countries (ranging from 1.6% in the Netherlands to 3.0% in Germany), with higher rates consistently reported for adolescents residing in Asia (i.e. 10.3% in mainland China) [2]. In China, the prevalence of IGD varied widely, ranging from 3.9% for high school students in Shanghai to 15.6% for secondary school students in Hong Kong [3, 4]. The discrepancies in prevalence rates of IGD have been largely attributed to measurement issues such as heterogeneity in assessment tools [5], or lack of measurement invariance across different groups. Such issues may confound accurate assessment of IGD prevalence, affecting the screening or identification of high-risk groups. Therefore, it is important for relevant instruments to be psychometrically evaluated in different populations.

The present study sought to address this aim by assessing the psychometric properties of the widely-used 7-item Game Addiction Scale (GAS) developed by Lemmens et al. [6]. The GAS was created in view of the substantial overlap between personality characteristics of gamblers and gaming addicts [6, 7], and has been used in various populations as an assessment tool to screen for IGD. Items on the GAS were adapted from the 7 diagnostic criteria (salience, tolerance, mood modification, withdrawal, relapse, conflict, and problems) for pathological gambling under DSM-IV-TR [6], and were confirmed as adequate to assess IGD among two independent samples of adolescents in Netherlands [6]. Concurrent validity of the GAS was found to be satisfactory, indicated by the correlations between scores on the GAS and time spent on games (*r* = 0.576), life satisfaction (*r* = − 0.136), loneliness (*r* = 0.314), social competence (*r* = − 0.158) and aggression (*r* = 0.265) [6]. Psychometric properties of the scale were later tested among gamers as well as the general population residing in France, Germany, Brazil, Spain, Iran and Italy [8–12]. Confirmatory factor analysis (CFA) showed that the scale had a unidimensional structure [9], with a Cronbach’s alpha value ranging from 0.85 [9] to 0.92 [10]. Although the GAS has been used to examine prevalence and correlates of IGD among adolescents and young adults in China [3, 13, 14], no study has focused on its psychometric properties in this population. Understanding and evaluating psychometric properties of the GAS in this population is an essential aspect of scale selection, it may enhance applicability of the scale and accuracy of the scale in identifying at-risk populations.

In the current study, we chose to assess psychometric properties of the GAS among the college student population in China for the following reasons: the adolescent population has been the main focus of existing studies, whereas the young adult population has been under-researched, as it is commonly perceived that, compared to other age groups, characteristics unique to adolescents may make them more vulnerable to developing IGD [15–18]. However, it is critical to note that adolescents in China typically face intense academic pressure due to fierce competitions in the college entrance exam, or Gaokao. In comparison, once students enter college in China, they are completely relieved of the academic pressure of Gaokao, and most likely divert their attention to other aspects of their college life [19]. Second, most adolescents live with their parents during their junior and high school years, when close proximity to parents can facilitate and strengthen parental monitoring. In comparison, many students choose to attend college away from their hometown and parents, a sign of independence, which may lead to decreased parental control and monitoring. A study examining health-related behaviors among middle school, high school and college students in China found screen time increased as educational level increased [20]. A recent study examining both high school and college students in China found college students scored higher on the IGD-20 Test [13].

Therefore, the purpose of this study was to assess the reliability and validity of the 7-item GAS using a sample of college students residing in China. We further assessed the association of IGD with mental health, sleep quality, substance use, problematic internet use, and social media addiction in establishing the validity of the GAS. Additionally, we sought to test and establish measurement invariance of the GAS across socio-demographic groups. Examining measurement invariance is an essential aspect of instrument validation, as it reflects the extent to which a measured construct has the same meaning across all respondents regardless of their group membership [21]. Findings of this study could expand the applicability of the 7-item GAS in assessing IGD to the Chinese college student population, and lay the ground work for further analysis and comparison.

## Methods

### Participants

A convenient sample of 1071 participants was recruited from multiple colleges in mainland China. Students who were attending school part-time or unable to complete the questionnaire were excluded, only full-time students who were willing to complete the questionnaire were included. We only included full-time students on the grounds that part-time or non-traditional students are usually older compared to traditional-age college students, and may enter college with work experience or family situations that can predispose them to a much different pattern of internet use behavior. As a result, respondents indicating they were graduate students (15, 1.40%) were excluded from final analysis. We checked the remaining data for missing values, and found 16 cases (1.49%) had missing values in the variable sleep efficiency, while all other cases had complete responses in every variable used in the analysis. As Schafer et al. suggested that a missing rate of 5% or less is commonly inconsequential [44], we performed complete case analysis. The final sample consisted of 1040 traditional-age college students, 416 of whom were males (40%) and 624 were females (60%). The maximum estimated sampling error of our sample was calculated to be ±3.04% with a 95% confidence probability [57].

### Measures

#### Internet gaming addiction (IGD)

IGD was measured by the Gaming Addiction Scale (GAS) developed by Lemmens et al. The GAS consists of seven Likert-type items (1 = never, 2 = rarely, 3 = sometimes, 4 = often, 5 = very often), which all begin with a statement “During the last 6 months, how often …” For example, “During the last 6 months, how often did you think about playing a game all day long?” Total score of the GAS is between 7 and 35, with higher scores indicating higher level of gaming addiction. Chinese version of the GAS was utilized to test IGD among adolescents, with a Chronbach’s alpha value between 0.93 and 0.94 [3]. Concurrent validity of the GAS has been confirmed by its correlation with Internet Addiction and hours of gaming among Italian adolescents [8]. Good internal reliability was reported in the present study (Chronbach’s alpha value = 0.951).

#### Problematic internet use

Problematic internet use was assessed by Young’s 20-item Internet Addiction Test (IAT) [58]. The scale was developed based on the diagnostic criteria for pathological gambling under the DSM-IV-TR. Each item is rated using a Likert scale (1 = never, 2 = rarely, 3 = sometimes, 4 = often, 5 = very often). For example, “How often do you find that you stay online longer than you intended?” The total score of the IAT is between 20 and 100. Chinese version of the IAT has demonstrated good internal consistency (Cronbach’s alpha = 0.93 [22];). Concurrent validity of the IAT has also been confirmed by its correlation with the Revised Chen Internet Addiction Scale (*r* = 0.46 [22];), the average online time per day (*r* = 0.40 for weekdays, *r* = 0.37 for weekends [22];), and the Mobile Phone Dependence Questionnaire (*r* = 0.59 [23];). Good internal reliability was reported in the present study (Chronbach’s alpha value = 0.938).

#### Sleep quality

Sleep quality was assessed using the Pittsburgh Sleep Quality Index (PSQI). The PSQI consists of 18 items that measure seven dimensions of sleep quality over the past month [24]: subjective sleep quality, sleep latency, sleep duration, habitual sleep efficiency, sleep disturbances, use of sleep medication, and daytime dysfunction. For example, “During the past month, how often have you had trouble sleeping because you have pain?” The total score of each dimension ranges from 0 to 3, with higher scores indicating poorer sleep quality. The total score of the whole scale is obtained by summing scores on each of the seven dimensions, ranging from 0 to 21. Chinese version of the PSQI has exhibited adequate internal consistency [25]. Consistent with values (0.62–0.66) reported in previous studies [25, 26], Cronbach’s alpha for the scale in the present study was 0.64 and considered to be acceptable. Composite reliability for the scale was 0.78, exceeding the recommended minimum value of 0.7 [49].

#### Psychiatric symptoms

Psychiatric symptoms were assessed using the Symptom Checklist 90-Revised (SCL-90-R) [27]. The SCL-90-R is a widely used self-report scale consisting of 90 items that examines nine symptomatic dimensions: somatization, obsessive-compulsiveness, interpersonal sensitivity, depression, anxiety, hostility, phobic anxiety, paranoid ideation, and psychoticism. An example of the items would be “How much were you bothered or distressed over the past 4 weeks by headaches?” The score of each item ranges from 0 to 5 (0 = not at all, 1 = a little bit, 2 = moderately, 3 = quite a bit, 4 = extremely), and score of each item is summed up to produce a total score between 0 and 360. Chinese version of the SCL-90-R exhibited good internal consistency (Cronbach’s α = 0.98) [28]. Scores on the nine subscales were significantly correlated with scores on the whole scale, indicating good structural validity [28, 29]. Criterion validity of the SCL-90-R has also been examined through its correlation with self-reported quality of life [28]. Cronbach’s alpha value for the scale was 0.977 in the current study.

#### Substance use

Substance use, including tobacco use, binge drinking and other drug use, were assessed within a 12-month time period. Tobacco use was assessed by asking whether respondents had used either traditional cigarettes or e-cigarettes. Binge drinking was assessed by asking whether respondents had at least had five drinks (including beer, wine, champagne and liquor) in one setting for males, and at least had four drinks in one setting for females. Other drug use was assessed by asking whether respondents had used marijuana, heroin, MDMA, sedatives, or over the counter (OTC) medications. Because the number of positive responses to each type of other drug use was relatively low, we combined responses to each type of other drug use into a single binary variable, comparing with those used at least one type of other drugs against those who answered “no” to all types of other drug use.

#### Social media addiction

Social media addiction was assessed by the Social Media Addiction Scale - Student Form (SMA-SF) developed by Sahin [30]. The scale consists of 29 items measuring 4 dimensions of social media addiction: virtual tolerance, virtual communication, virtual problem and virtual information. Total score of the SMA-SF ranges from 29 to 145, with higher scores indicating higher levels of social media addiction [30]. Each item can be rated on a 5-point Likert-type scale (1 = Definitely not appropriate, 2 = Not appropriate, 3 = Undecided, 4 = Appropriate, 5 = Quite appropriate) In the original study, the SMA-SF exhibited good internal reliability (Cronbach’s alpha = 0.93), split-half reliability (Guttmann Split-Half value = 0.90) and test-retest reliability (test-retest coefficient = 0.94). In this study, the SMA-SF demonstrated good internal reliability, the value of Cronbach’s alpha for the scale was calculated to be 0.955.

### Procedure

We used a popular professional online survey platform (https://www.wjx.cn/) in China to prepare and present the survey. Recruitment occurred between June to August, 2019. The link to the survey was distributed via Wechat messages. All participants were informed that participation was completely anonymous and that their responses would be kept confidential. Upon completion of the survey, each participant was given 2 Chinese yuan (about $0.3 USD).

### Statistical analysis

All analyses were conducted using SPSS 22.0 and AMOS 24.0. Reliability of the scale was assessed by internal consistency and split-half reliability. Validity of the scale was assessed by structural validity, convergent validity, discriminant validity, and concurrent validity.

#### Reliability

Internal consistency represents the extent to which different items are correlated, and was assessed using Cronbach’s alpha coefficients, theta coefficient and omega coefficient [31]. A coefficient of greater than 0.7 indicates good internal consistency [32]. Split-half reliability indicates stability of the scale, and was measured using the Spearman-Brown coefficient, with higher values representing higher stability [33].

#### Validity

Exploratory factor analysis (EPA) was conducted to examine the factor structure of the GAS. Previous studies have found the 7-item GAS to have a unidimentional structure [6]. Confirmatory factor analysis (CFA) was used to measure structural validity of the GAS. The goodness-of-fit of the model was examined using a series of indices: the χ^2^ to degrees of freedom ratio (χ^2^ / df), comparative fit index (CFI), goodness of fit index (GFI) and root mean square error of approximation (RMSEA). The assessment criteria for each index were: χ^2^ / df < 3, CFI > 0.9, GFI > 0.9 and RMSEA< 0.08 [34].

Convergent validity of the scale was measured by the value of average variance extracted (AVE), which was calculated using a formula $\frac{\sum \left(\mathrm{factor}\kern0.17em \mathrm{loading}\kern0.17em \mathrm{value}\right)2}{\sum \left(\mathrm{factor}\kern0.17em \mathrm{loading}\kern0.17em \mathrm{value}\right)2+\sum \left(\mathrm{measurement}\kern0.17em \mathrm{error}\right)}$. The convergent validity of a scale is considered acceptable if the value of AVE is higher than 0.50 [35]. Concurrent validity was measured by the association between the GAS and the IAT, PSQI, SCL-90 and substance use. The Pearson product-moment correlation coefficient was used to assess their associations. The correlation coefficient ranges between − 1.0 and 1.0, an absolute value of ≥0*.*5 is considered large, an absolute value between 0.3 and 0.5 is considered moderate, an absolute value between 0.1 and 0.3 is considered small, and an absolute value less than 0.1 is considered trivial [36]. Discriminant validity refers to whether dissimilar constructs can be differentiated, and was measured by the correlation between GAS and SMA-SF in the present study. A Pearson’s value of less than 0.85 indicates adequate discriminant validity [35].

Multigroup confirmatory factor analysis (MCFA) was conducted to test the measurement invariance of the GAS across gender, class standing, income and parental educational level. Three nested models were adopted: 1) a configural model (model 1), in which all factor parameters were freely estimated; 2) a weak factorial invariance model (model 2), in which item loadings were constrained to be equal across groups; and 3) a strict factorial invariance model (model 3), in which item residuals were constrained to be equal across groups. Chen [37] recommends that measurement invariance is not supported if CFI decreases by a value greater than 0.01 or RMSEA increases by a value greater than 0.015 [37]. Because the GAS is an ordinal scale, maximum likelihood estimation may not be the appropriate estimate, asymptotically distribution-free estimation was used to accommodate non-normally distributed data in SEM analyses instead.

### Ethics

The study procedures were carried out according to the Declaration of Helsinki. The Institutional Review Board of the [Name of the Institution] approved this study. All participants were informed about the study, and all provided informed consent.

## Results

Sample characteristic of the final 1040 respondents (416 male and 624 female) were shown in Table 1.

**Table 1:** Sample characteristics

Characteristics	Total (*n* = 1040)
Gender	
Male	416 (40%)
Female	624 (60%)
Class standing	
Freshmen	264 (25.4%)
Sophomores	491 (47.2%)
Juniors & Seniors	285 (27.4%)
Family income	
< 50,000	241 (23.2%)
50,000 ~ 100,000	309 (29.7%)
50,000 ~ 200,000	302 (29.0%)
> 200,000	188 (18.1%)
Father’s educational level	
≤ Middle school	381 (36.3%)
High school	258 (24.8%)
≥ College	401 (38.6%)
Mother’s educational level	
≤ Middle school	436 (41.9%)
High school	250 (24.0%)
≥ College	354 (34.0%)
Internet gaming disorder	16.41 (7.07)
Problematic internet use	54.09 (16.29)
Sleep quality	5.45 (2.92)
Psychological symptom	
Interpersonal sensitivity	6.38 (7.36)
Depression	8.81 (10.56)
Anxiety	5.58 (7.58)
Hostility	3.40 (4.64)
Phobic anxiety	3.35 (5.24)
Paranoid ideation	3.31 (4.59)
Phychoticism	5.62 (7.62)
Socia media addiction	81.29 (22.77)
Substance use	
Past-year tobacco use	179 (17.2%)
Past-year binge drinking	276 (26.5%)
Past-year substance use	304 (29.2%)

Note: Values are presented as mean (SD) or number (percentage) when appropriate

### Reliability

The GAS exhibited satisfactory internal consistency and split-half reliability, with Cronbach’s alpha value of 0.951, theta coefficient value of 0.953, omega coefficient value of 0.959, and a Spearman-Brown coefficient value of 0.938. All the items demonstrated good corrected item-total correlations, ranging from 0.781 to 0.867 (Table 2).

**Table 2:** Corrected item-total correlation and reliability indices

Item	Construct	Corrected item-total correlation
item1	Salience	0.849
item2	Tolerance	0.781
item3	Mood modification	0.855
item4	Relapse	0.867
item5	Withdrawal	0.835
item6	Conflict	0.830
item7	Problems	0.841
Chronbach α		0.951
Theta coefficient		0.953
Omega coefficient		0.959
Spearman-Brown Coefficient		0.938

### Validity

#### Structural validity, convergent validity and discriminant validity

EFA revealed a one-factor model of the GAS, which was further confirmed by CFA. The model exhibited satisfactory fit indices: χ^2^ / df = 0.877 (*p* < 0.05), CFI = 0.999, GFI = 0.996, RMSEA = 0.000 (90% CI = 0.000, 0.035). In addition, no standardized factor loading was below 0.76 (Table 3). The GAS exhibited good convergent and discriminant validity, with the AVE value to be 0.734 and the value of Pearson’s correlation coefficient to be 0.520 (Table 3).

**Table 3:** Standardized factor loading, goodness-of-fit indices, convergent and discriminant validity indices

Item	Construct	Factor loading
item1	Salience	0.86
item2	Tolerance	0.76
item3	Mood modification	0.89
item4	Relapse	0.88
item5	Withdrawal	0.87
item6	Conflict	0.87
item7	Problems	0.86
χ2 /df		0.877
CFI		0.999
GFI		0.996
RMSEA		0.000
AVE		0.734
Pearson’r		0.520

#### Concurrent validity

As shown in Table 4, correlation between the GAS total score and the IAT total score was large (*r* = 0.672). Correlation between the GAS total score and the SCL-90-R total score (*r* = 0.455) and subscale scores was moderate, somatization (*r* = 0.483), obsessive-compulsive symptoms (*r* = 0.382), interpersonal sensitivity (*r* = 0.390), depression (*r* = 0.414), anxiety (*r* = 0.440), hostility (*r* = 0.457), phobic anxiety (*r* = 0.467), paranoid ideation (*r* = 0.457), and psychoticism (*r* = 0.427). Correlation between the GAS total score and substance use total score was also moderate (*r* = 0.367). However, the correlation of GAS total score with PSQI total score was small (*r* = 0.220).

**Table 4:** Correlations between GAS and other constructs

	1	2	3	4	5	6	7	8	9	10	11	12	13
1.GAS	1.00	0.67	0.22	0.48	0.38	0.39	0.41	0.44	0.46	0.47	0.46	0.43	0.37
2.IAT		1.00	0.31	0.43	0.45	0.43	0.45	0.43	0.43	0.42	0.43	0.41	0.26
3.PSQI			1.00	0.42	0.48	0.44	0.47	0.45	0.42	0.39	0.42	0.41	0.20
4.Somatization (subscale)				1.00	0.82	0.82	0.86	0.91	0.87	0.90	0.88	0.87	0.61
5.Obsessive-compulsiveness (subscale)					1.00	0.91	0.91	0.88	0.84	0.82	0.85	0.86	0.43
6.interpersonal sensitivity (subscale)						1.00	0.93	0.90	0.88	0.85	0.90	0.90	0.46
7.Depression (subscale)							1.00	0.93	0.88	0.87	0.89	0.91	0.49
8.Anxiety (subscale)								1.00	0.92	0.91	0.92	0.93	0.55
9.Hostility (subscale)									1.00	0.89	0.91	0.89	0.55
10.Phobic anxiety (subscale)										1.00	0.89	0.89	0.59
11.Paranoid ideation (subscale)											1.00	0.92	0.55
12.Psychoticism (subscale)												1.00	0.57
13.Substance use													1.00

Note: *GAS* Gaming Addiction Scale, *IAT* Internet Addiction Test, *PSQI* Pittsburgh Sleep Quality Index, *SCL-90-R* Symptom Checklist-90-Revised

### Measurement invariance

Model fit indices across gender, class standing, family income and parental educational level are presented in Table 5. Results indicated that the GAS had strict measurement invariance across educational level of father and mother respectively, supported by the acceptance of model 2 and model 3. Model fit indices including Δ**χ**2 (df), ΔCFI and ΔRMSEA are presented in Table 6. For gender, values of model fit indices were Δ **χ**2 (df) = 14.910 (6), ΔCFI = − 0.013, ΔRMSEA = 0.014 for weak factorial model, and Δ **χ**2 (df) = 105.666 (21), ΔCFI = − 0.120, ΔRMSEA = 0.041 for strict factorial model. For class standing, values of model fit indices were Δ**χ**2 (df) = 26.129 (12), ΔCFI = − 0.016, ΔRMSEA = 0.019 for weak factorial model, and Δ**χ**2 (df) = 72.809 (42), Δ CFI = − 0.037, ΔRMSEA = 0.021 for strict factorial model. For family income, values of model fit indices were Δ **χ**2 (df) = 21.76 (12), ΔCFI = − 0.011, ΔRMSEA = 0.005 for weak factorial model, and Δ **χ**2 (df) = 78.121 (42), ΔCFI = − 0.042, ΔRMSEA = 0.010 for strict factorial model. Results of model 2 and 3 revealed that the GAS exhibited no weak or strict measurement invariance across gender, class standing and family income.

**Table 5:** Factor loading and model fit across gender, class standing, family income and parental educational level

	Factor loading							Model fit			
Item	Item1	Item2	Item3	Item4	Item5	Item6	Item7	χ2 /df	CFI	GFI	RMSEA
Gender											
Male	0.80	0.72	0.87	0.85	0.83	0.84	0.85	0.714	1.000	0.996	0.000
Female	0.90	0.8	0.91	0.90	0.89	0.87	0.88	1.411	0.99	0.983	0.026
Class standing											
Freshmen	0.90	0.75	0.93	0.87	0.92	0.86	0.98	1.469	0.981	0.976	0.042
Sophomores	0.85	0.76	0.89	0.88	0.84	0.89	0.83	0.537	1.000	0.994	0.000
Juniors & Seniors	0.85	0.84	0.87	0.91	0.88	0.84	0.90	0.764	1.000	0.993	0.000
Family income											
< 50,000	0.856	0.773	0.93	0.931	0.89	0.919	0.897	0.898	1.000	0.987	0.000
50,000 ~ 100,000	0.859	0.785	0.887	0.834	0.914	0.85	0.874	1.179	0.995	0.985	0.024
50,000 ~ 200,000	0.871	0.825	0.881	0.878	0.854	0.844	0.873	0.939	1.000	0.986	0.000
> 200,000	0.855	0.77	0.884	0.893	0.898	0.879	0.876	1.825	0.973	0.972	0.066
Father’s educational level											
≤ Middle school	0.877	0.818	0.899	0.911	0.894	0.9	0.852	1.037	0.999	0.990	0.010
High school	0.856	0.724	0.925	0.894	0.851	0.869	0.905	1.072	0.998	0.987	0.010
≥ College	0.845	0.81	0.871	0.851	0.876	0.831	0.867	0.981	1.000	0.985	0.000
Mother’s educational level											
≤ Middle school	0.894	0.789	0.902	0.908	0.894	0.887	0.894	1.836	0.986	0.985	0.044
High school	0.871	0.814	0.896	0.842	0.867	0.894	0.829	1.041	0.999	0.985	0.013
≥ College	0.886	0.816	0.879	0.902	0.886	0.765	0.842	1.279	0.989	0.978	0.028

**Table 6:** Measurement invariance across gender, class standing, family income and parental educational level

Model		Model Fit Indices					
		χ2 (df)	***Δ***χ2 (Δdf)	CFI	ΔCFI	RMSEA	ΔRMSEA
Gender	Configural	14.876 (14)		0.999		0.008	
	Weak factorial	29.786 (20)	14.910 (6)	0.986	−0.013	0.022	0.014
	Strict factorial	120.542 (35)	105.666 (21)	0.879	−0.120	0.049	0.041
Class standing	Configural	19.403 (21)		1.000		0.000	
	Weak factorial	45.532 (33)	26.129 (12)	0.984	−0.016	0.019	0.019
	Strict factorial	92.212 (63)	72.809 (42)	0.963	−0.037	0.021	0.021
Family income	Configural	55.048 (49)		0.993		0.011	
	Weak factorial	76.808 (61)	21.76 (12)	0.982	−0.011	0.016	0.005
	Strict factorial	133.169 (91)	78.121 (42)	0.951	−0.042	0.021	0.010
Education(F)	Configural	21.631 (21)		0.999		0.005	
	Weak factorial	40.759 (33)	19.128 (12)	0.990	−0.009	0.015	0.010
	Strict factorial	71.74 (63)	50.109 (42)	0.989	−0.010	0.012	0.007
Education(M)	Configural	29.091 (21)		0.990		0.019	
	Weak factorial	35.77 (33)	6.679 (12)	0.997	0.007	0.009	−0.010
	Strict factorial	78.222 (63)	49.131 (42)	0.981	−0.009	0.015	−0.004

Considering the rejection of weak measurement invariance across gender, class standing and family income, partial invariance for each item was further examined. For gender, after checking the result of measurement invariance, item 2 had the largest value. So the loading of item 2 was set to vary and the weak measurement invariance was tested again. Values of model fit indices were Δ **χ**2 (df) = 6.027 (5), ΔCFI = − 0.002, ΔRMSEA = 0.002, indicating that partial invariance was supported for gender when the loading of item 2 was set to vary. The same process of setting free the loading of the item with the largest measurement invariance until |ΔCFI| < 0.01 and ΔRMSEA < 0.015 was repeated for class standing and family income. As shown in Table 7, the non-invariant factors were salience, mood modification, relapse, withdrawal, conflict and problems for gender; salience, tolerance, mood modification, relapse, withdrawal and conflict for class standing; salience, tolerance, mood modification, relapse, conflict and problems for family income.

**Table 7:** Partial measurement invariance across gender, class standing and family income

Model	Model Fit Indices					
Gender						
	**χ**2 (df)	***Δ*χ**2 (Δdf)	CFI	ΔCFI	RMSEA	ΔRMSEA
Model 1.1	14.876 (14)		0.999		0.008	
Model 1.2	29.786 (20)	14.910 (6)	0.986	−0.013	0.022	0.014
Model 1.3	20.903 (19)	6.027 (5)	0.997	−0.001	0.010	0.002
Class standing						
Model 2.1	19.403 (21)		1.000		0.000	
Model 2.2	45.532 (33)	26.129 (12)	0.984	−0.016	0.019	0.019
Model 2.3	32.775 (31)	13.372 (10)	0.998	−0.002	0.007	0.007
Family income						
Model 3.1	55.048 (49)		0.993		0.011	
Model 3.2	76.808 (61)	21.76 (12)	0.982	−0.011	0.016	0.005
Model 3.3	67.005 (59)		0.991	−0.002	0.011	0

Model 1.1: Unconstrained model

Model 1.2: All item loading equal

Model 1.3: item loadings 1,3,4,5,6,7 equal

Model 2.1: Unconstrained model

Model 2.2: All item loading equal

Model 2.3: item loadings 1,2,3,4,5,6 equal

Model 3.1: Unconstrained model

Model 3.2: All item loading equal

Model 3.3: item loadings 1,2,3,4,6,7 equal

## Discussion

This study is the first to examine the psychometric properties and measurement invariance of the 7-item GAS among Chinese college students. Consistent with results from previous studies, [9–11], we found the GAS had a unidimensional structure and exhibited excellent reliability. Our findings on measurement invariance of the GAS across different socio-demographic groups lent support to existing studies that found measurement invariance of the GAS across linguistic groups [9], gender and groups spending different amounts of time on gaming [11]. More specifically, we found the GAS had strict measurement invariance across parental educational levels, suggesting that scores on the GAS reflected respondents’ gaming behaviors rather than the influence of their parents’ level of education. We also found partial measurement invariance was supported for gender, class standing and family income groups. That is, all items except for tolerance were found to be operating equivalently across gender; all items except for problems were found to be operating equivalently across class standing; all items except for withdrawal were found to be operating equivalently across family income. According to a previous study, the imprecision of the concept of withdrawal may cause unexplained variance in different groups [9]. Our study further revealed the relative weakness of the items of problems and tolerance when assessing internet gaming addiction among the Chinese college student population.

Our results indicated a moderate association between the scores on the GAS and the total as well as subscale scores on the SCL-90-R. This finding was consistent with previous studies reporting the association between IGD and subscales of SCL-90-R such as depression, anxiety, somatization [38–40], interpersonal sensitivity, obsessive-compulsiveness, phobic anxiety, hostility [40], psychoticism and overall severity [41]. Although gaming may be a way to cope with psychological distress, yet, excessive gaming can result in elevated levels of depression, anxiety and social phobia [42]. Moreover, excessive gaming may lead to increased risk of exposure to violent games, as gaming addicts seemed to have more normative beliefs about aggressions and to engage in more hostile behaviors [42]. The overlap between IGD and obsessive-compulsiveness may be attributed to impairment in inhibitory control, which may lead to repetitive dysfunctional behaviors [43]. Excessive gaming was also associated with reduced motivation in other social activities, which could result in subsequent interpersonal problems [45].

We found a moderate association between the total score on the GAS and substance use. This finding lent support to previous findings on the positive association between IGD and alcohol, tobacco, and illicit drug use [46–48]. Substance use has been found to be a common comorbidity of Internet addiction, as those with substance use disorder seemed to exhibit similar core symptoms of IGD [50, 51]. Both substance use disorder and IGD have been associated with deficient reward system functions, manifested as having higher responsiveness to substances and video games and lower responsiveness to other natural rewards as a result of altered dopamine levels. Another shared mechanism of these two types of addictive behaviors involves high trait impulsivity. Individuals with high trait impulsivity tend to perform poorly on decision-making tasks, focusing on short-term consequences instead, thus giving priority to addictive behaviors rather than other behaviors [50, 52].

In regards to IGD and sleep quality, some studies found their association to be significant [53]. It is plausible that some gamers may become deprived of sleep due to significant amount of time spent playing games, or report daytime sleepiness as a consequence [54]. Some studies even showed that delayed sleep phase can improve by readjusting individual circadian rhythm with exogenous day-light cycle, thus alleviating gaming-related sleep problems [55]. However, a systematic review study by Lam found insufficient evidence supporting a strong association between IGD and poor sleep quality [56], but found a stronger association between problematic internet use and sleep problems. In line with Lam’s review study, we found the association between gaming addictions and sleep quality to be smaller than the association between problematic internet use and sleep quality, implying that differing mechanisms may be involved in how playing internet games or engaging in excessive internet use relays to sleep quality. Although investing these mechanisms is beyond the aim of this study, future studies are needed to examine the underlying mechanisms contributing to these differences.

Previous studies have indicated that gaming addiction was associated with less conscientiousness and low openness, while social networking addiction was associated with high neuroticism and extraversion [14], suggesting that gaming addiction and social networking addiction may be associated with differing personality traits. In the present study, the relatively small correlation between GAS and SMA-SF scores suggested that the GAS can discriminate gamers from people with other types of Internet-related addictive behaviors such as social media addiction.

### Strengths and limitations

To our knowledge, this is the first study to assess the psychometric properties of the 7-item GAS among the college student population in China. Findings of this study provided ample support for the application of the GAS as a screening tool to assess IGD among this population. However, this study also has several limitations that we would like to acknowledge along with prospective directions for future research. First, this study mainly utilized self-report data such as on sleep quality and substance use, reporting or recalling biases might have affected the accuracy of the testing results. Future studies may need to incorporate more objective measures. Second, the cross-sectional nature of our data limited us to draw tentative conclusions about the temporal sequence of IGD development. Longitudinal studies may be needed to clarify this sequence. Third, our study mainly focused on Chinese college students, our findings may not be applicable to same-age populations in other countries. More studies from other countries to corroborate our findings of the GAS.

## Conclusions

This study entails that the 7-item GAS is a reliable and valid instrument for assessing IGD among Chinese college students, ensuring researchers and clinicians that it is an adequate tool to examine problematic gaming.

## Data Availability

The datasets used and/or analyzed during the current study are available from the corresponding author on reasonable request.

## Abbreviations

IGD: Internet Gaming Disorder
GAS: Gaming Addiction Scale
PIU: Problematic Internet Use
MG-CFA: Multigroup Confirmatory Factor Analysis
